# 23例肺癌并发肺栓塞的临床分析

**DOI:** 10.3779/j.issn.1009-3419.2014.03.12

**Published:** 2014-03-20

**Authors:** 娜 李, 燕 王

**Affiliations:** 1 300052 天津，天津医科大学总医院呼吸科 Department of Respiration Medicine, Tianjin Medical University General Hospital, Tianjin 300052, China; 2 300052 天津，天津医科大学总医院肿瘤内科 Department of Medical Oncology, Tianjin Medical University General Hospital, Tianjin 300052, China

**Keywords:** 肺肿瘤, 肺栓塞, 化疗, 生存时间, Lung neoplasms, Pulmonary thromboembolism, Chemotherapy, Survival time

## Abstract

**背景与目的:**

已有研究结果表明肺栓塞是恶性肿瘤的常见并发症，并以肺癌多见，本研究探讨肺癌并发肺栓塞患者的临床特点、危险因素及对生存期的影响。

**方法:**

回顾性分析2009年6月-2013年7月在天津医科大学总医院肿瘤内科就诊明确为肺癌并确诊为合并肺栓塞的23例患者的临床资料，对照组为随机选取同期在总医院肿瘤内科确诊为肺癌患者46例，采用*Kaplan*-*Meier*分析总生存时间。

**结果:**

共确诊肺癌1, 128例，其中23例明确诊断肺栓塞，肺癌合并肺栓塞患者腺癌16例（69.6%），不明原因呼吸困难16例（69.6%），较肺癌组高（*P* < 0.01），肺癌合并肺栓塞组的血红蛋白、白蛋白、血氧分压较肺癌组低（*P* < 0.05），白细胞、D-Dimer较肺癌组高（*P* < 0.05），两组血小板、谷丙转氨酶、谷草转氨酶无统计学差异。肺癌合并肺栓塞同时合并下肢深静脉血栓患者10例（43.48%），急性肺栓塞高危患者6例（26.09%）。单因素分析显示，血红蛋白 < 100 g/L、白细胞 > 11×10^9^/L、D-Dimer > 500 ng/mL、血氧分压 < 80 mmHg、白蛋白 < 30 g/L是肺癌发生肺栓塞的危险因素（OR值分别为5.50、11.03、4.83、4.68、9.63）。16例（69.6%）肺栓塞分布于肺癌确诊前后5个月内，肺栓塞发生于确诊肺癌的时间中位数为3.05个月。截止到2013年7月29日，肺癌合并肺栓塞组患者的中位生存时间为7.77个月，明显低于肺癌组患者的19.27个月（*P*=0.02），肺癌合并肺栓塞患者化疗组生存时间高于非化疗组。

**结论:**

肺癌合并肺栓塞患者的常见病理类型为腺癌，其临床表现常见不明原因呼吸困难、咳嗽，肺癌确诊后前5个月为肺栓塞高发时期，肺癌患者发生肺栓塞会降低其生存时间。化疗对于肺癌合并肺栓塞患者的生存有益。

## Abstract (en)

**Background and objective:**

Malignant tumors are often complicated with pulmonary thromboembolism (PTE), particularly in lung cancer (LC). This study investigated the clinical characteristics, risk factors, and survival time of patients with PTE and LC.

**Methods:**

We retrospectively reviewed the clinical data of patients with LC in Medical Oncology of Tianjin Medical University General Hospital between June 2009 and July 2013. A total of 23 patients suffered from both PTE and LC. A total of 46 cases were used as control subjects. The survival of these patients was compared with that of the control subjects by Kaplan-Meier analysis.

**Results:**

A total of 1128 cases were diagnosed with LC, in which 23 were clearly diagnosed with PTE, and 16 (69.6%) patients who suffered from both LC and PTE developed adenocarcinoma. These patients also exhibited a higher incidence of unexplained dyspnea than those who were diagnosed with LC only (*P* < 0.01). The patients with both PTE and LC displayed lower counts of hemoglobin (Hb), albumin (ALB), and blood oxygen pressure (PO_2_) than those with LC only (*P* < 0.05). The patients with both PTE and LC also exhibited higher counts of leukocyte (WBC) and D-dimer than those with LC only (*P* < 0.05). No statistical difference was found between the two groups in terms of platelet counts, glutamic pyruvic transaminase level, and glutamic oxaloacetic transaminase level (*P* > 0.05). Ten cases (43.48%) with deep venous thrombosis were observed in the PTE and LC group, and six patients (26.09%) were at a high risk of acute pulmonary embolism. Hb < 100 g/L, WBC > 11×10^9^/L, D-Dimer > 500 ng/ml, PO_2_ < 80 mmHg, ALB < 30 g/L were the risk factors of LC with PTE (odds ratio values were listed as follows: 5.50, 11.03, 4.83, 4.68, 9.63, respectively). On July 29, 2013, the median survival time of patients with PTE and LC was 7.77 months. This result was significantly lower than that (19.27 months) of patients with LC only (*P*=0.02). The survival time of patients suffering from both LC and PTE and undergoing chemotherapy was higher than that of patients who did not undergo such treatment.

**Conclusion:**

The common pathological type observed in patients with both LC and PTE was adenocarcinoma. The common clinical manifestations were unexplained dyspnea and cough. The first five months after LC diagnosis corresponded to a high period of PTE. Patients with LC and PTE exhibited less survival time. As such, chemotherapy is beneficial for the survival of patients with LC and PTE.

静脉血栓栓塞症（venous thromboembolism, VTE）包括深静脉血栓形成（deep venous thrombosis, DVT）和肺血栓栓塞症（pulmonary thromboembolism, PTE），是恶性肿瘤患者的常见并发症。早在1865年Trousseau就发现恶性肿瘤与静脉血栓栓塞症存在着联系，研究^[1, 2]^显示肺癌发生静脉血栓的风险是普通人群的22倍。大部分研究关于恶性肿瘤发生血栓事件，而肺癌合并肺栓塞的研究较少，本研究旨在对我院肿瘤内科住院患者中23例肺癌合并肺栓塞患者的临床特点进行分析，探讨其临床特征、危险因素、生存时间，提高对本病的认识。

## 1 对象与方法

### 1.1 研究对象

收集于2009年6月-2013年7月在天津医科大学总医院肿瘤内科住院治疗的1, 128例肺癌患者的临床资料，均经病理学明确诊断肺癌，其中疑诊及确诊肺栓塞患者79例（7.0%），确诊肺癌合并肺栓塞患者中资料完整者23例，对照组患者为随机选取同期入院肺癌患者46例，采用性别，治疗方式匹配。

### 1.2 诊断标准

PTE的诊断按中华医学会呼吸病学分会制定的《肺血栓栓塞症诊断和治疗指南》（草案）诊断标准^[3]^；急性肺栓塞诊断标准按2008 ESC（欧洲心脏病学会）急性肺栓塞诊治指南^[4]^；按照UICC（国际抗癌联盟）2009年修订的肺癌分期进行分期。对照组46例患者根据诊断标准证实无肺栓塞及血栓性疾病。

### 1.3 资料采集

采集患者临床资料及实验室参数如下：性别、年龄、吸烟史、饮酒史、基础疾病、既往治疗史；血细胞计数、血生化肝肾功能、肿瘤标记物、病理类型及分期、心电图、下肢血管超声、确诊肺栓塞影像学依据、末次随访时间及死亡时间。采用电话方式随访，均无失访，随访时间至患者死亡时间或2013年7月29日为止，存活时间以月为单位。

### 1.4 统计学处理

采用SPSS 17.0进行数据统计分析，计量资料，服从正态分布的应用Mean±SD表示，两组间的比较采用独立样本*t*检验，不服从正态分布的采用M±Q表示，组间率的比较采用非参数检验，计数资料以率或构成比表示，两组间率的比较采用卡方检验。采用*Logistic*进行单因素危险分析检验。采用*Kaplan*-*Meier*计算患者的总生存时间。*P*＜0.05为差异具有统计学意义。

## 2 结果

### 2.1 患者一般资料

肺癌合并肺栓塞组患者：23例，平均年龄67.26±8.34岁，男/女：13/10，其中腺癌16例（69.6%），鳞癌4例（17.4%），小细胞肺癌3例（13.0%），分期Ⅰ期+Ⅱ期4例（17.4%），Ⅲ期+Ⅳ期19例（82.6%）。

肺癌组患者：46例，平均年龄63.52±6.85岁，男/女：26/20，腺癌23例（50.0%），鳞癌13例（28.3%），小细胞肺癌10例（21.7%）。分期Ⅰ期+Ⅱ期14例（30.4%），Ⅲ期+Ⅳ期32例（69.6%）（表 1）。

**1:** 肺癌伴/不伴肺栓塞患者的一般临床资料 Clinical characteristics of LC patients with/without PTE

Clinical characteristic	LC with PTE (*n*=23)	LC without PTE (*n*=46)	*P*
Mean age (year)	67.26	63.52	0.05
Male gender	13 (56.5%)	26 (56.5%)	> 0.99
Histological type			
Adenocarcinoma	16 (69.6%)	23 (50.0%)	0.20
Squamous cell carcinoma	4 (17.4%)	13 (28.3%)	0.55
Small cell carcinoma	3 (13.0%)	10 (21.7%)	0.74
Stage			
Ⅰ-Ⅱ	4 (17.4%)	14 (30.4%)	0.39
Ⅲ-Ⅳ	19 (82.6%)	32 (69.6%)	0.38
LC: lung cancer; PTE: pulmonary thromboembolism.			

### 2.2 临床症状及体征

肺癌合并肺栓塞组患者：咳嗽、不明原因呼吸困难为最常见临床症状，其次为咯血、心悸，肺栓塞三联征发生率低，不明原因呼吸困难及烦躁不安较肺癌组患者发生率高（表 2）。肺癌合并肺栓塞患者首发表现，最常见的症状为呼吸困难47.83%，其次为发热、胸痛，双肺呼吸音粗、呼吸音低为最常见体征。

**2:** 肺癌伴/不伴肺栓塞患者的临床表现 Clinical manifestation of LC patients with/without PTE

Clinical manifestation	LC with PTE (%)	LC without PTE (%)	*P*
Asymptomatic	3 (13.0)	8 (17.4)	> 0.99
Cough	18 (78.3)	29 (63.0)	0.09
Unexplained dyspnea	16 (69.6)	12 (26.1)	< 0.01
Chest pain	5 (21.7)	3 (6.5)	0.10
Syncope	1 (4.3)	2 (4.3)	> 0.99
Dysphoria	4 (17.4)	1 (2.2)	0.03
Panic	1 (4.3)	1 (2.2)	0.53
Impending death	2 (8.7)	1 (2.2)	0.23
Hemoptysis	7 (30.4)	15 (32.6)	0.58
Palpitation	6 (26.1)	6 (13.0)	0.17
PTE three union syndrome	2 (8.7)	0 (0)	0.10

### 2.3 实验室检查及影像学检查

① 血气分析：在未吸氧的情况下，肺癌合并肺栓塞组患者，以PO_2_＜80 mmHg患者多见，与肺癌组患者比较，肺癌合并肺栓塞组患者低氧更多见。②肺癌合并肺栓塞组患者与肺癌组患者的实验室结果比较，肺癌合并肺栓塞组的血红蛋白（hemoglobin, Hb）、白蛋白（albumin, ALB）、血氧分压（blood oxygen pressure, PO_2_）较肺癌组低（*P*＜0.05），白细胞（leukocyte, WBC）、D-Dimer较肺癌组高（*P*＜0.05），两组血小板（platelet, PLT）、谷丙转氨酶（glutamic-pyruvic transaminase, ALT）及谷草转氨酶（glutamic oxalacetic transaminase, AST）无统计学差异（*P*＞0.05）（表 3，表 4）。③肺癌合并肺栓塞患者心电图表现窦性心动过速4例（17.39%），SIQⅢTⅢ（Ⅰ导联见宽大的S波，Ⅲ导联出现Q波、T波倒置）患者3例（13.0%），电轴右偏，肺型P波2例（8.7%），胸导联T波倒置2例（8.7%），RBBB（右束支传导阻滞）1例（4.35%），合并下肢深静脉血栓患者10例（43.48%）。④肺癌合并肺栓塞患者影像学检查，22例患者胸部强化CT提示肺动脉内的密度充盈缺损。1例患者肺通气血流灌注扫描提示双肺多发血流灌注明显减低。急性肺栓塞高危患者6例（26.09%），低危及中危患者17例（73.91%）。

**3:** 肺癌伴/不伴肺栓塞实验室指标 Laboratory index of patients in LC with/without PTE

Laboratory index	LC with PTE			LC without PTE		*t*	*P*
	*n*	Mean±SD		*n*	Mean ±SD		
RBC	23	4.05±0.64		46	4.29±0.57	1.61	0.11
Hb	23	107.87±21.09		46	128.46±18.33	4.18	< 0.01
PLT	23	246.35±91.03		46	240.70±74.01	-0.28	0.78
ALB	23	31.86±4.4		46	37.43±3.98	5.03	< 0.01
RBC: erythrocyte; Hb: hemoglobin; PLT: blood platelet; ALB: albumin.							

**4:** 肺癌伴/不伴肺栓塞实验室指标 Laboratory index of patients in LC with/without PTE

Laboratory index	LC with PTE			LC without PTE		*Z*	*P*
	*n*	M±Q		*n*	M±Q		
WBC	23	9.00±5.2		46	6.80±2.24	-2.89	< 0.01
CEA	23	17.47±42.66		46	13.97±15.74	-0.69	0.49
D-Dimer	23	2100±3300		46	166.50±634	-4.31	< 0.01
PO_2_	23	63.00±25		46	84.00±17.00	-4.05	< 0.01
ALT	23	20.00±26.25		46	17.50±10.50	-0.71	0.48
AST	23	18.00±18.50		46	18.50±12.00	-1.24	0.21
WBC: leukocyte; CEA: carcino embryonie antigen; PO2: blood oxygen pressure; ALT: glutamic-pyruvic transaminase; AST: glutamic oxalacetic transaminase.							

### 2.4 肺栓塞距离肺癌确诊时间

23例肺癌合并肺栓塞患者中，2例（8.7%）肺栓塞发生在肺癌确诊之前，21例（91.3%）发生肺癌确诊后，其中最长时间为52.6个月，16例（69.57%）肺栓塞分布于肺癌确诊前后5个月内，肺栓塞发生于确诊肺癌的时间中位数为3.05个月。3例（13.04%）肺癌合并肺栓塞患者于肺栓塞后24 h内死亡，均为急性肺栓塞高危分层患者，11例（47.82%）于肺栓塞后1月内死亡，最长生存时间为12.4个月。

### 2.5 单因素分析肺癌发生肺栓塞危险因素

纳入肺癌伴发肺栓塞的危险因素主要有：基础疾病、生活方式、治疗方案、病理类型、肿瘤分期、实验室指标。模型Hb＜100 g/L，CEA＞10 ng/mL，WBC＞11×10^9^/L，PO_2_＜80 mm/Hg，D-Dimer＞500 ng/mL。

结果发现腺癌栓塞风险增加了1.5倍，Ⅲ期-Ⅳ期肺癌及接受化疗患者栓塞风险增加了1倍以上，此外还发现低血红蛋白血症、高白细胞血症、高D-Dimer、低氧血症及低蛋白血症患者发生肺栓塞的风险增加（OR值分别为5.50、11.03、4.83、4.68、9.63），余指标无统计学差异（表 5）。

**5:** 单因素分析肺癌合并肺栓塞危险因素 Risk factors related to PTE of LC by univarite analysis

	LC with PTE	LC without PTE	OR	95%CI	X^2^	*P*
Adenocarcinoma	16	23	2.10	0.27-3.75	1.94	0.16
Stage Ⅲ-Ⅳ	19	32	0.25	0.22-3.27	0.05	0.82
Chemotherapy	17	28	2.38	0.80-7.13	2.54	0.11
Disbetes	4	8	1.00	0.27-3.35	0.00	> 0.99
COPD	7	14	0.84	0.30-2.32	0.12	0.80
Smoking	9	20	0.78	0.28-2.19	0.28	0.42
Alcohol drinking	3	6	0.90	0.20-4.00	0.02	> 0.99
Hemoglobin < 100 g/L	11	5	5.50	1.67-18.08	8.66	0.01
CEA > 10 ng/mL	13	30	0.69	0.25-1.93	0.49	0.48
WBC > 11×10^9^/L	10	3	11.03	2.63-46.15	13.11	< 0.01
D-Dimer > 500 ng/mL	17	17	4.83	1.60-14.62	8.68	0.007
PO_2_ < 80 mm/Hg	18	20	4.68	1.48-14.77	5.75	0.01
Albumin < 30 g/L	7	2	9.63	1.81-51.25	8.71	0.01
PD: chronic obstructive pulmonary disease.						

### 2.6 肺癌合并肺栓塞与肺癌组患者的生存分析

肺癌合并肺栓塞组患者的中位生存时间为7.77个月，肺癌组患者生存时间为19.27个月。对两组患者进行*Kaplan*-*Meier*生存比较，肺癌合并肺栓塞组总生存时间明显低于肺癌组（*P*=0.02）（图 1A）。

**1:**
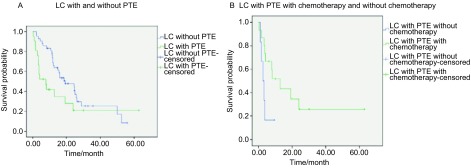
生存曲线。A：肺癌合并肺栓塞与肺癌组生存分析（*P*=0.02）；B：肺癌合并肺栓塞化疗与未化疗生存分析（*P*=0.033）。 Survival curve. A: survival analysis beween LC with and without PTE (*P*=0.02); B: survival analysis beween LC with PTE with chemotherapy and without chemotherapy (*P*=0.033).

### 2.7 治疗与转归

肺癌合并肺栓塞全部患者均经低分子肝素、华法林抗凝治疗，3例经溶栓治疗。高危肺栓塞患者死亡6例，中危及低危肺栓塞患者死亡10例。将肺癌合并肺栓塞患者根据治疗方法分为化疗组，非化疗组，对总生存时间进行*Kaplan*-*Meier*生存比较，发现化疗组生存时间高于非化疗组（*P*＜0.05）（图 1B）。

## 3 讨论

肺血栓栓塞是由来自右心或静脉系统的血栓阻塞肺动脉干及其分支而引发肺循环以及呼吸功能障碍的一系列病理生理变化的临床综合征，往往导致致死或致慢性肺动脉高压，研究^[5]^显示未经治疗的急性肺栓塞患者病死率高达25%-30%，VTE是恶性肿瘤常见并发症及第二大死亡原因。因此，了解肺癌合并肺栓塞的临床表现，动态实验室检查和危险因素尤为重要。

本研究中肺癌合并肺栓塞患者最常见临床表现为咳嗽、不明原因呼吸困难、咯血，这些症状虽然不是肺栓塞所特有的，但不明原因呼吸困难、烦躁不安明显高于肺癌组，因此肺癌患者出现上述症状要高度警惕肺栓塞。

根据Khorana设计的肿瘤患者VTE发生率的评分标准^[6]^，分析肺癌合并肺栓塞的危险因素，本组研究中，高白细胞血症的肺癌患者继发肺栓塞的风险增高达10倍，可能机制为白细胞激活血小板和血管内皮细胞，并同时与这两种细胞相互作用，分泌组织因子及血管内皮生长因子，促进血栓形成^[7]^。本研究中Hb < 100 g/L的患者发生肺栓塞的风险增加4.5倍，欧洲癌症贫血调查组研究肺癌贫血发生率为77%，且有研究^[8]^显示Hb升高是肺癌合并肺栓塞的危险因素，因此血红蛋白作为肺癌患者预测肺栓塞的指标有待进一步研究。许多研究显示D-Dimer诊断PTE的阴性预测值较高，敏感性差，可作为PTE的排除标准，本组研究中D-Dimer > 500 ng/mL肺癌患者发生肺栓塞风险增加3.8倍，D-Dimer可作为肺癌患者预测肺栓塞的指标^[9]^。本组研究中动态观察肺栓塞前后心电图变化出现3例SIQⅢTⅢ（13.0%），其次有胸导联T波倒置，RBBB，有研究显示，肺栓塞动态心电图表现可表现为房性心律失常、RBBB、ST段压低、ST段抬高、异常Q波和肢体导联低电压，警惕临床工作者应注意肺癌患者心电图变化，提高肺栓塞诊断意识。

本组研究中肺腺癌16例（69.6%）发生肺栓塞，肺腺癌患者发生肺栓塞风险增加1.1倍，与研究相符，原因可能为肺腺癌细胞产生的组织蛋白酶激活机体凝血系统，并分泌粘液蛋白，引起机体变态反应，使血管内膜及周围组织退变、纤维素样变和上皮细胞脱落，造成局部血栓形成，肿瘤粘蛋白可单独激活血小板并产生经典的弥漫性凝血引发Trousseau综合征^[10, 11]^。

研究显示肺栓塞可能是恶性肿瘤的首发症状，4%-12%的患者VTE确诊伴随恶性肿瘤，本研究中16例（69.57%）肺栓塞分布于肺癌确诊前后5个月内，其中2例肺栓塞为肺癌的首发症状，与研究相符，VTE在肿瘤确诊的最初3个月内发病率最高，约增加53倍，随后发病率降低^[2]^。肿瘤引起机体体液变化激活了凝血系统，同时初期肿瘤治疗会激发其高凝状态。

本组研究中，肺癌合并肺栓塞组患者13例接受过含铂两药化疗药物，3例接受过靶向药物治疗，肺癌患者化疗发生肺栓塞风险增加1.38倍，有文献^[12]^报道恶性肿瘤患者系统化疗后发生深静脉血栓的概率是普通人群的2倍-6倍。原因可能是化疗、激素、靶向药物治疗可破坏肿瘤细胞诱发急性肿瘤溶解综合征，可加重患者高凝状态引发血栓。肺癌合并肺栓塞化疗患者的生存时间较肺癌患者高，说明抗肿瘤治疗虽增加肺癌合并肺栓塞肺癌患者的肺栓塞风险，但是对于肺癌患者的生存仍有益。

本研究中肺癌合并肺栓塞组患者生存时间明显低于肺癌组患者，9例患者（42.9%）于肺栓塞确诊后1月内死亡，与Chuang研究^[10]^相符，需要进一步研究肺癌合并肺栓塞后治疗对其生存期的影响。肺癌患者一旦发生肺栓塞，导致其生存期缩短，治疗难度增加，治疗费用增高，后果严重，因此早期诊断及预防显得尤为重要。本研究有很多局限性，一方面入组患者较少，不能反映总体肺癌合并肺栓塞患者的临床特点，另一方面，对照组肺癌患者亚临床静脉栓不能排除。总之，肺癌患者出现不明原因呼吸困难、烦躁不安时及时行实验室及影像学检查，提高肺栓塞的诊断意识。
